# PROTOCOL: Effects of virtual reality exposure therapy versus in vivo exposure in treating social anxiety disorder in adults: A systematic review and meta‐analysis

**DOI:** 10.1002/cl2.1259

**Published:** 2022-06-30

**Authors:** Martin Polak, Norbert Tanzer, Per Carlbring

**Affiliations:** ^1^ Department of Psychology University of Graz Graz Austria; ^2^ Department of Psychology Stockholm University Stockholm Sweden

## Abstract

This is the protocol for a Campbell systematic review. The objectives are as follows: (a) to quantify the effect sizes for virtual reality exposure therapy (VRET) in the treatment of social anxiety disorder (SAD), targeting primary social anxiety symptoms, comorbid anxiety and depression symptoms and improvements in quality of life, when compared to WL, information control, care‐as‐usual and placebo; (b) to compare VRET to in vivo cognitive and cognitive‐behavioral interventions in treating SAD, at posttest and follow‐up, using between‐group design; (c) to identify the key features which are linked to beneficial outcomes in the two formats in treating SAD and (d) to collect and interpret information on differences in treatment uptake, adherence and attrition, as well as clinical significance and therapist‐time in both treatment formats.

## 1 BACKGROUND

In addition to conventional face‐to‐face cognitive‐behavioral therapy (CBT) interventions, virtual reality exposure therapy (VRET) presents an innovative and promising intervention with similar efficacy in the treatment of social anxiety disorder (SAD). The main objective of the current study is to compare the efficacy of VRET with various in vivo behavioral and cognitive‐behavioral interventions, in selected randomized controlled trials (RCTs). Systematic literature search will include electronic searches, as well as searching through gray literature and engaging in personal and e‐mail communication with experts in the field. This study will investigate the efficacy of VRET compared to in vivo exposure in treating SAD, at posttest and follow‐up using between‐group design. Another aim is to identify the key features which are linked to beneficial outcomes in the two formats in treating SAD. Information on differences in treatment uptake, adherence, and attrition, as well as clinical significance and therapist‐time in both treatment formats will be collected in interpreted. We will quantify effect sizes for VRET in the treatment of SAD, targeting primary social anxiety symptoms, comorbid anxiety and depression symptoms, and improvements in quality of life, when compared to inactive controls such as wait‐list, information control, care‐as‐usual, and placebo.

### 1.1 Description of the condition

#### 1.1.1 SAD

SAD is a severe anxiety disorder with lifetime prevalence of 10.3% for women and 8.7% for men (McLean et al., 2011). It affects different aspects of a person's life such as social activities, relationships, career, and academic functioning (American Psychiatric Association [APA], 2013). Characteristic symptoms are related to the physical, cognitive, and behavioral features with most prominent factors being fear of scrutiny and negative evaluation from others that leads to feelings of embarrassment, humiliation, and shame (Brunello et al., 2000). Post‐event rumination and safety‐behaviors along with self‐focused attention play a crucial role in the maintenance of SAD (Clark & Wells, 1995). A clinical diagnosis requires that individuals’ anxiety has to be out of proportion—in frequency and/or duration—to the actual situation, with significant distress or impairment that interferes with ordinary routine in social settings (APA, 2013). There are two main diagnostic categories for SAD. A non‐generalized form of SAD is related to one aspect of social functioning, most commonly associated with performance‐type social activities (Angélico et al., 2013), afflicting approximately 40% of all those who suffer from social phobia (Ruscio et al., 2008). On the other hand, a generalized form of SAD affects at least 50% of affected individuals (Furmark et al., 2000; Vriends et al., 2007). It is associated with more psychiatric comorbidities and functional impairment (Fink et al., 2009; Heimberg et al., 2000). In general, there is a high frequency of psychiatric comorbidity in patients with SAD, occurring in as many as 90% of affected individuals (Acarturk et al., 2008; Ohayon & Schatzberg, 2010; D. J. Stein et al., 2017).

### 1.2 Description of the intervention

#### 1.2.1 Psychological treatment of SAD

CBT is considered as “gold standard” among psychological treatments for anxiety disorders, particularly for its comprehensive scientific evidence base and superiority over alternative therapies such as psychodynamic therapy (Tolin, 2010).

In treating of SAD, traditional delivery of CBT proves to be effective not only in reducing primary social anxiety symptoms (Acarturk et al., 2009; Fogarty et al., 2019; Hofmann & Smits, 2008; Mayo‐Wilson et al., 2014), but also in comorbid anxiety or depression (Acarturk et al., 2009; Fogarty et al., 2019; Fracalanza et al., 2014) and improvements in quality of life (Watanabe et al., 2010). In addition, meta‐analyses have documented its positive long‐term effects (Fogarty et al., 2019; van Dis et al., 2020). Although there are various CBT treatment programs for SAD (Clark & Wells, 1995; Heimberg & Becker, 2002; Hope et al., 2006), the common denominator is identifying and changing maladaptive beliefs about physical symptoms and their consequences, conceptualizing avoidance behavior as the maintaining factor of SAD as well as exposure to anxiety‐provoking stimuli.

In SAD, treatment‐seeking behavior is generally rare, delayed, and often accompanied by another psychiatric disorder (Acarturk et al., 2008; M. B. Stein, 2006). According to Kessler and Greenberg (2002), approximately two‐thirds of individuals affected by any anxiety disorder remain untreated, reporting high treatment costs as one of the most common reasons for not entering psychological treatment. Also, long travel distances for people living in rural areas make the probability of entering a traditional face‐to‐face psychotherapy lower (Shapiro, Cavanagh & Lomas, 2003). As only about one‐third of individuals suffering from anxiety receive treatment (Kazdin, 2015), there is a great demand for an innovation in the treatment of this mental health condition.

Cognitive behavioral therapy provided over the Internet (iCBT) is becoming increasingly common, particularly when it is supported by a mental health professional. In treating of SAD, iCBT was found effective at both short and long term in a number of meta‐analyses (El Alaoui et al., 2015; Hedman et al., 2011).

Unlike traditional CBT or iCBT, where affected individuals are motivated to confront the feared stimuli in real life, VRET present a highly effective treatment intervention for a broad spectrum of mental health conditions. It uses the innovative technology of virtual reality, which enables confrontation to feared stimuli in a variety of virtual environments. In addition to changing unproductive thinking and conceptualizing avoidant behavior, VRET treatment protocols include gradual exposure to stimuli that cause anxiety and disrupt normal functioning of afftected individuals in their everyday life (Parker et al., 2018). The method of VRET offers various advantages to traditional exposure delivery, such as that during exposure, clients can interact with virtual stimuli in a safe and controlled environment, which makes it easier to get used to the feared stimuli. As such, VRET present a platform where effective relaxation training can be easily incorporated (Schultheis & Rizzo, 2001). In virtual reality, the whole exposure process can be repeated as many times as necessary, which clearly is a major advantage over conventional exposure interventions. Examples from real‐life practice show us that when trying to confront feared stimuli in vivo, it is usually difficult to repeat the exposure as much as needed within a short period of time. As one example, we present aviophobia, where individuals affected by excessive fear of flying usually experience difficulties practicing multiple exposures in vivo, as air travel still is a costly and logistically demanding mode of transportation. In the literature, the process of getting used to such feared stimuli is known as systematic desensitization, and its use is most often found in the treatment of phobias (Kaplan & Tolin, 2011).

### 1.3 How the intervention might work

#### 1.3.1 VRET

VRET uses computer‐generated environments providing input to the user's sensory system. Visual virtual stimuli are usually presented via virtual reality (VR) glasses, also known as head mounted display (HMD). In addition, auditory input is applied via loudspeakers or earphones. It is also possible to provide a tactile, haptic or olfactory stimulation, however, this is seldom the case in clinical trials. The overall aim of VR is to replace sensory input from the real world and to create a presence of the user in the virtual world. To interact with the user in real time, the VR system collects information about the users' position and (head) movements via sensors and input devices like a head tracking system or a joystick (Diemer et al., 2015).

When compared to psychological placebos and wait‐lists (WL), VRET is a highly effective treatment intervention with effects generalized to real life (Morina et al., 2015, Powers & Emmelkamp, 2008) and a low deterioration rate (Fernández‐Álvarez et al., 2019). It represents a cost‐efficient approach (Wood et al., 2009), highly preferred among young individuals (Garcia‐Palacios et al., 2001) or individuals affected by specific phobias (Garcia‐Palacios et al., 2007). Moreover, when compared to in vivo exposure in treatment of anxiety disorders, VRET has found to be equally effective on the short‐ and long‐term (Carl et al., 2019; Fodor et al., 2018; Powers & Emmelkamp, 2008). However, despite number of meta‐analyses confirming the high effectiveness of VRET in treating anxiety disorders (Carl et al., 2019; Fodor et al., 2018; Meyerbröker & Emmelkamp, 2010; Opriş et al., 2011; Parsons & Rizzo, 2008; Wechsler et al., 2019), the effect of VRET in treating SAD seems to be more complex.

Whereas meta‐analyses from Carl et al. (2019), Chesham et al. (2018), Fodor et al. (2018), Kampmann et al. (2016) found VRET to be equally effective as the traditional face‐to‐face CBT with in vivo exposure in the treatment of SAD, findings from Wechsler et al. (2019) suggest the opposite. This meta‐analysis found a significant effect size favoring in vivo exposure (*g* = −0.50) over VRET.

As a result, previous meta‐analyses differ in their findings with regard to the efficacy of VRET versus in vivo exposure in treating SAD, with quality of design and methodology of included studies varying significantly.

### 1.4 Why it is important to do this review

Previous meta‐analyses use mixed samples with clinical and nonclinical levels of social anxiety, as well as RCTs and quasi‐randomized trials. It seems that when investigating the efficacy of VRET, clinical effectiveness is derived from the studies with predominantly small samples, with substantial risk of bias, and by incomplete reporting (Georgescu et al., 2019; Page & Coxon, 2016). Furthermore, at least four previous meta‐analyses on this topic (Carl et al., 2019; Chesham et al., 2018; Fodor et al., 2018; Kampmann et al., 2016) include either incomplete data or duplicate the same data in their meta‐analytical calculations. These meta‐analyses include preliminary results (Robillard et al., 2010) and final results (Bouchard et al., 2011) of the same study, published as separate conference abstracts. This certainly contributes to results ambiguity in this research topic. Previous meta‐analyses on this topic also vary considerably in investigating and interpreting of treatment uptake and adherence, clinical significance, treatment attrition, and changes in comorbid symptoms. Although the majority of previous meta‐analyses confirm an equal efficacy of VRET in SAD when compared to in vivo exposure, results from the recent meta‐analysis from Wechsler et al. (2019) contradict these findings. Here, authors investigated the efficacy of VRET compared to in vivo exposure in treating specific phobias, agoraphobia, and SAD, however, there were only three RCTs included. In light of these findings, there is a demand for a new, methodologically rigorous meta‐analysis investigating the efficacy of VRET in SAD. Our study follows the methodology of registered reports to secure the most advanced methodology using peer review process to align scientific values and practices. The aim of this study is to compare VRET to in vivo exposure in treating SAD, at posttest and follow‐up using between‐group design and to identify the key features which are linked to beneficial outcomes in the two formats in treating SAD. Also, we will quantify effect sizes for VRET in the treatment of SAD, targeting primary social anxiety symptoms, comorbid anxiety and depression symptoms and improvements in quality of life, when compared to inactive controls such as WL, information control, care‐as‐usual, and placebo.

## 2 OBJECTIVES

The objectives of the current study are (a) to quantify the effect sizes for VRET in the treatment of SAD, targeting primary social anxiety symptoms, comorbid anxiety, and depression symptoms, and improvements in quality of life, when compared to WL, information control, care‐as‐usual and placebo; (b) to compare VRET to in vivo cognitive and cognitive‐behavioral interventions in treating SAD, at posttest and follow‐up, using between‐group design; (c) to identify the key features which are linked to beneficial outcomes in the two formats in treating SAD and (d) to collect and interpret information on differences in treatment uptake, adherence, and attrition, as well as clinical significance and therapist‐time in both treatment formats.

## 3 METHODS

### 3.1 Criteria for considering studies for this review

#### 3.1.1 Types of studies

All included studies will be RCT.

We will formulate additional exclusion criteria: (a) non‐RCTs, pilot studies, open trials and feasibility studies; (b) trials with samples including less than 10 subjects per experimental group, as this was also the case in the previous studies; (c) trials with SAD being secondary diagnosis; (d) trials with internet‐based CBT interventions for SAD or PSA; (e) studies written in a language other than English and German.

#### 3.1.2 Types of participants

All included studies will include participants who met the DSM‐IV or DSM‐5 criteria for SAD or public speaking anxiety (PSA). Changes in symptoms will be investigated in an adult population.

#### 3.1.3 Types of interventions

All studies will have to include at least one virtual reality exposure session designed for treating SAD or PSA.

#### 3.1.4 Types of outcome measures

All included studies will use at least one clinician‐administered rating or behavioral assessment test. All studies will use valid and reliable measures for assessing levels of social anxiety, comorbid anxiety and depression and improvements in quality of life.

##### 3.1.4.1 Primary outcomes

Social anxiety symptoms, comorbid anxiety and depression and improvements in quality of life.

##### 3.1.4.2 Secondary outcomes

Information on differences in treatment uptake, adherence and attrition, as well as clinical significance and therapist‐time.

### 3.2 Search methods for identification of studies

To find eligible individual studies for our meta‐analytical calculations, we will adopt the recommendations from Lipsey and Wilson (2001) and proceed as follows.

#### 3.2.1 Electronic searches

First, we will conduct an electronic computer search through databases such as PsycINFO, PubMed and Web of Science. For German‐language studies, we will search through the PSYNDEX database, using German equivalents.

We will search for the keywords “virtual” and “phobia” in the PubMed, PsychInfo, and Web of Science databases. Moreover, we will conduct a search on the term “social anxiety” and “public speaking anxiety.” We will not set a time limit for the period in which the studies were conducted. Depending on the different databases' search template structure, we will use different search strategies accordingly.

In the PsycInfo database, we will search for “virtual” in title and “phobia” in abstracts, as well as for “virtual reality” in title and “anxiety” and “social” and "public speaking" in abstracts. In PubMed, the connector ‘AND' will be used to search for “Virtual AND Phobia”, as well as “Virtual AND anxiety AND social” and “Virtual AND anxiety AND public speaking” in titles and abstracts. In Web of Science, we will search for “virtual” in title and “phobia” in the topic, as well as for “virtual” in title and “anxiety” and “social” and "public speaking" in the topic.

Subsequent searches will be made in the Cochrane Central Register of Controlled Trials (CENTRAL), ClinicalTrials.gov, and PROSPERO, where titles and abstracts of relevant studies will be investigated.

#### 3.2.2 Searching other resources

Second, we will search through so‐called “gray literature” and examine abstracts of conference contributions and posters, using Scopus and CENTRAL. To further identify potentially relevant studies, we will screen reference lists of the found literature (snowball search). Additionally, we will search for government documents, using general internet search engines such as Google and Google Scholar to identify potential published and unpublished studies, as well as dissertations and theses (Kugley et al., 2017). For the latter, we will use the PsycInfo database as well. To ensure picking up the most current information, we will perform web searches toward the end of the search phase of a review. When using web search engines, search strategies will be entered into the Advanced search screen, as this allows to easily use Boolean logic and limiting commands through the use of menus (Kugley et al., 2017). Keywords such as “randomized controlled trial” and “virtual reality exposure therapy” and “social anxiety” will be used to limit the results to empirical research. For searches in the German language, we will use German equivalents.

Third, we will engage in personal and e‐mail communication with experts in the field, to get access to their (unpublished) articles regarding this topic. This will be done individually, as well as through mailing lists.

The searches will be repeated several times, until February 2022.

### 3.3 Data collection and analysis

Information on sample sizes, means, standard deviations, and standardized mean differences will be transferred to Microsoft Excel spreadsheet and then to Comprehensive Meta‐Analysis (version 3.0; Biostat, Inc.).

#### 3.3.1 Selection of studies

The first author (MP) will initially screen all titles and abstracts to determine their relevance to this paper. Studies that can be immediately excluded based on the title and abstract will be discarded. All remaining records with no evidence for a violation in eligibility criteria within their abstracts will be passed on for full‐text screening. Both the first and second author will independently review remaining studies for inclusion eligibility.

#### 3.3.2 Data extraction and management

The following data will be extracted from each study: authors and year of publication; type of assignment; diagnosis with subtype (if presented); sample type and number of participants before allocation; treatment type; control condition; virtual environments; modules/therapist‐time per patient; type of clinician rating; outcome scales (primary, secondary and tertiary); type of statistical analysis; outcome points; therapist experience and country.

#### 3.3.3 Assessment of risk of bias in included studies

Both the first and the second author will independently assess risk of bias in the included studies. In agreement with the RoB 2: A revised Cochrane risk‐of‐bias tool for randomized trials (Sterne et al., 2005), five default areas of potential risk of bias will be investigated. As VRET interventions cannot be blinded from the clinicians' point of view, we will not include blinding of participants and personnel. Hence, the included studies will be assessed for: (1) bias arising from the randomization process; (2) bias due to deviations from intended interventions; (3) bias due to missing outcome data; (4) bias in measurement of the outcome; (5) bias in selection of the reported result. We will interpret all areas in terms of low, high, or some concerns of risk of bias. If the risk of bias will be rated as high or some concerns in more than three domains, a study will be rated with an overall high risk. All differences will be discussed and reconciled.

#### 3.3.4 Measures of treatment effect

For the metric outcome measures, we will calculate effect size Hedges' *g*, which is a bias‐adjusted estimate of the standardized mean difference particularly eligible for trials with small samples. Hedges’ *g* represents the difference between means of a treatment intervention and comparison condition, divided by the pooled standard deviation (Hedges, 1981). Positive values of *g* (with the 95% confidence interval) indicate superiority of treatment condition over control condition. Effect sizes of 0.2, 0.5, and 0.8 will be considered small, moderate, and large respectively (Cohen, 1977). For clinical significance, we will use the risk difference as effect size (Borenstein et al., 2009). In case the included studies will differ in their outcome measures, we will transform the different effect size matrics into Hedges’ *g* to facilitate a single analysis.

#### 3.3.5 Unit of analysis issues

We expect individually RCTs with participants randomly allocated to intervention or control groups.

#### 3.3.6 Dealing with missing data

We will explore the possibility of publication bias using funnel plot and trim‐and‐fill analysis (Duval & Tweedie, 2000).

#### 3.3.7 Assessment of heterogeneity

We will examine heterogeneity using the *Q*‐statistic. Here, we will consider the proposition from Borenstein et al. and set the level of significance to *p* < 0.05, indicating presence of heterogeneity. Furthermore, the *I* ^2^‐index will be used in estimating of the observed variance proportion that reflects true differences in effect sizes between the studies. We will interpret the heterogeneity values of 25%, 50%, and 75% as low, moderate, and high, respectively (Crombie & Davies, 2009). In case a moderate to high heterogeneity will complicate the interpretation of mean effect sizes, a moderator analysis will be performed (Crombie & Davies, 2009).

#### 3.3.8 Assessment of reporting biases

Funnel plots will illustrate each effect size plotted against its standard error. An asymmetrical funnel plot will have a statistically significant test confirming reporting biases. To further test the asymmetry of the funnel plot, an Egger's asymmetry test of the intercept will be reported with a *p* > 0.05 significance level (Egger et al., 1997). If publication bias is suspected, then a “trim and fill” method will adjust for symmetry by the iterative addition and removal of studies to gauge the impact on the overall effect. In case there will be missing information within the included studies, we will opt for contacting the authors directly.

### 3.4 Data synthesis

To compute an effect size across studies, we will use random effects model (REM) as it assumes that a treatment effect in each study is randomly selected from a normal distribution and that it varies from study to study (Borenstein et al., 2009). Each statistical analysis will include a mean effect size with 95% confidence interval and a heterogeneity analysis, which will assess the degree of dispersion of the effect sizes around the mean effect (Hedges & Olkin, 1985; Rothstein et al., 2005).

### 3.5 Subgroup analysis and investigation of heterogeneity

To explore possible moderators of treatment effect in the VRET group, subgroup analyses will be used in categories such as (a) type of assessment scale (LSAS or pooled effect sizes from all scales targeting SAD symptoms); (b) sample size (*N* > or <30); (c) country of original research, (d) adherence to the treatment defined as initial uptake (low or high percentage of login to the treatment program), and (e) baseline severity (score higher or lower as 30 points on the LSAS scale).

In case there will be a sufficient number of studies included in our meta‐analysis (*k* > 10) (Higgins et al., 2021), we will perform a meta‐regression to guard against confounding.

### 3.6 Sensitivity analysis

We will conduct a sensitivity analysis of study peculiarities during the search and coding process to ascertain the robustness of the results.

### 3.7 Summary of findings and assessment of the certainty of the evidence

Collected and yielded data will be summarized in several tables holding information on the following: (1) changes in symptoms in form of effect sizes (primary measures) in VRET compared to inactive controls at posttest and follow‐up; (2) changes in symptoms in form of effect sizes (primary measures) in VRET compared to active controls at posttest and follow‐up; (3) benchmark effect size estimates of available treatments for SAD; (4) baseline and posttest estimates from LSAS scale in VRET and active controls.

## CONTRIBUTIONS OF AUTHORS

- Content: MP, NT, PC
- Systematic review methods: MP, NT, PC
- Statistical analysis: MP, NT
- Information retrieval: MP, NT

## DECLARATIONS OF INTEREST

MP is founder of VIRTUO, a start‐up specializing in VRET for anxiety disorders. At the time of the title registration with CSR and determination of the structure of the current systematic review, the company was not yet founded. PC has published several peer‐reviewed papers in the field.

## PRELIMINARY TIMEFRAME

Approximate date for submission of the systematic review: April 2022.

## DIFFERENCES BETWEEN PROTOCOL AND REVIEW

**Characteristics of studies**

**Characteristics of included studies**

***New Study 1***

**Table:** 

**Methods**
**Participants**
**Interventions**
**Outcomes**
**Notes**

Risk of bias table

**Table:** 

Bias	Authors' judgment	Support for judgment
Random sequence generation (selection bias)	Unclear risk	
Allocation concealment (selection bias)	Unclear risk	
Blinding of participants and personnel (performance bias)	Unclear risk	
Blinding of outcome assessment (detection bias)	Unclear risk	
Incomplete outcome data (attrition bias)	Unclear risk	
Selective reporting (reporting bias)	Unclear risk	
Other bias	Unclear risk	

## SOURCES OF SUPPORT

**Internal sources**

• No sources of support provided

**External sources**

• No sources of support provided
